# The evolution of cochlear implant technology and its clinical relevance

**Published:** 2014

**Authors:** M Hainarosie, V Zainea, R Hainarosie

**Affiliations:** * ”Carol Davila” University of Medicine and Pharmacy, Bucharest, Romania; ** ”Prof. Dr. Dorin Hociota” Institute of Phonoaudiology and Functional ENT Surgery, Bucharest, Romania

**Keywords:** cochlear implant, sound processor, electrode array

## Abstract

The article presents a brief history of the development of the cochlear implant, from its beginnings to the present day. After a short description of the device, it describes the evolution of the technology for three of the top manufacturing companies, from the first model marketed, to the latest. It presents the technological advancements from one model to the next, taking into account the exterior design, processing capabilities and functionality.

## Background

**Fig. 1:**
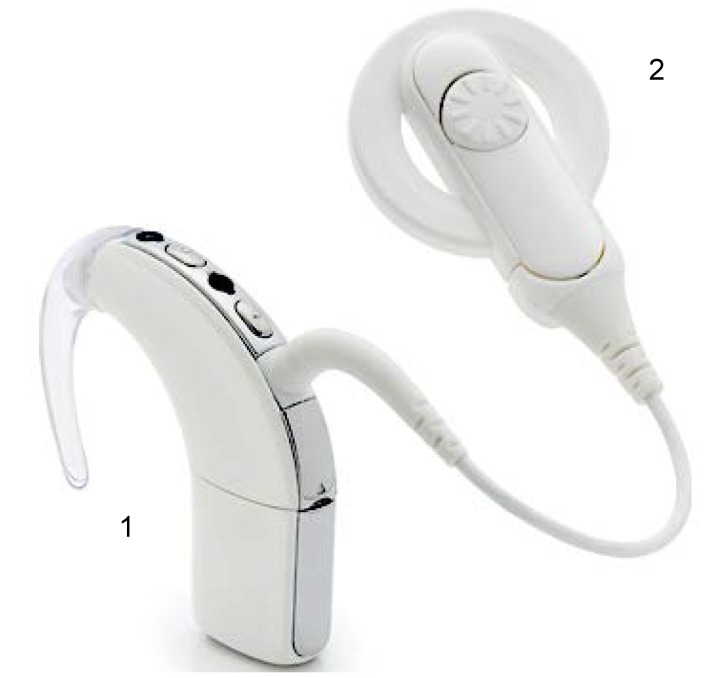
External component of a cochlear implant: (1) behind the ear sound processor; (2) antenna with magnet

**Fig. 2:**
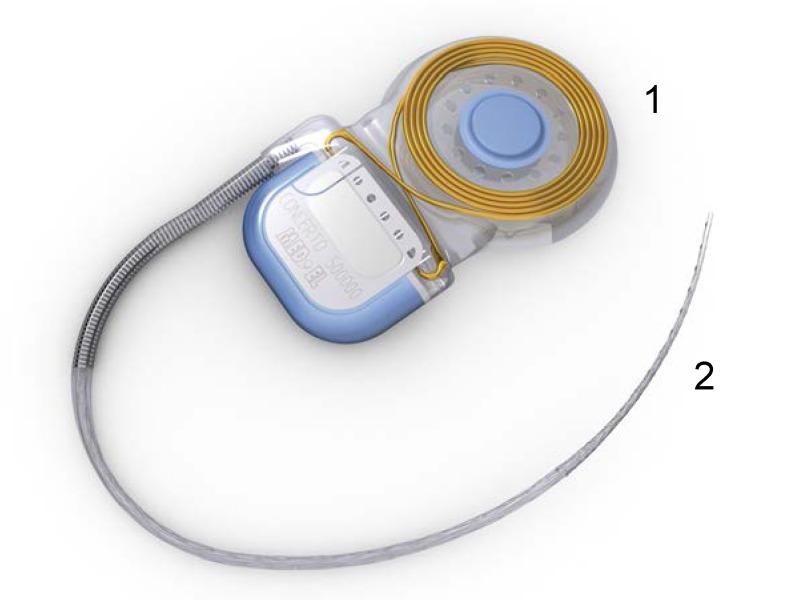
Internal component of a cochlear implant: (1) receiver-stimulator with coil and magnet; (2) intracochlear electrode array

Today, a cochlear implant is considered a reliable way to restore hearing in patients with severe to profound hearing loss and has been an approved method of treatment since the mid-1980s. A cochlear implant system consists of internal and external components. The internal system is surgically implanted inside the auditory system while the external system is worn behind the ear, in a pocket, in a belt pouch, or with a harness. The external system consists of a microphone, a speech processor, a transmitter, and a magnet. Its role is to capture the sounds from the environment, to process them, transforming the auditory signals in electrical signals and to transmit them to the internal part. The internal system consists of a receiver, stimulator, electrode system, and magnet. Its role is to receive the electrical signals from the outer part and to transmit them to the auditory nerve fibers in the cochlea. It sustains the external part through its magnet. **Fig. 1** shows an example of the external component. **Fig. 2** shows an example of the internal component.

The development of the cochlear implant started with the discovery that the electrical current could convey meaningful sounds to the brain. Unlike typical hearing aids, cochlear implants are constituted of an external device and a surgically implantable part. Also, hearing aids simply amplify sounds to enhance sound perception, whereas cochlear implants bypass the outer and middle ear to electrically stimulate the acoustic nerves within the auditory system. After more than two decades of research and development, cochlear implants have evolved from single channel devices to multichannel cochlear implant systems. Additionally, according to their design, technological developments have yielded substantial gains in the possibility of communication, development of language skills, spoken words recognition and media enjoyment for the users.

**A Brief History**

An overview of the beginnings of the cochlear implant is necessary to better understand the slow progression and breakthroughs that led to today’s technology.

In 1800, Alessandro Volta managed to stimulate his internal ear by using electrical current. 

In 1957, André Djourno and Charles Eyriès, the first one a professor of medical physics and the second one a Parisian otologist, tried to restore the facial nerve function of a patient affected by extensive bilateral cholesteatoma, which left him also deaf, by using a wire with electrical current. The function of the facial nerve was not restored, but the patient experienced auditory sensations [**1**].

In 1961, William House, an otologist from Los Angeles, and John Doyle, a neurosurgeon, developed an electrode that they placed through the round window in two patients. These reported auditory percepts, also noticing the change of loudness when the level of stimulation varied and the change of the pitch with the variation in the rate of stimulation. It took House ten years to develop a single channel device. This, the 3M/House device, was first implanted in 1972 and continued to be used until 1985 [**2**].

In the meantime, Graeme Clark, an ENT professor from Melbourne, gathered, in 1967, a team to conduct basic research on the pathophysiology of profound deafness in animals and on the tolerability of implanted materials. His efforts materialized by the introduction, in 1984, by the Cochlear Company, of the first multichannel cochlear implant system [**3**,**4**].

In Europe, Kurt Burian, an Austrian ENT professor, launched, in 1975, his own research and development of a single- than a multichannel device. His work was continued by his pupil, Ingeborg Hochmair and her husband Erwin Hochmair, in Innsbruck. Their work culminated in 1982 with the launch of the MedEl implant [**5**].

**Cochlear Implant Systems**

There are four cochlear implant manufacturers: Cochlear Limited (Australia), MedEl Corporation (Austria), Advanced Bionics (USA, a division of Sonova) and Neurelec (France, a division of William Demant). Only the first three are FDA-approved for the use in the United States. Neurelec is used in Europe, Asia, Africa, South America and Canada. Lately, a fifth manufacturer, Nurotron from China, has been available in some parts of the world. In Romania, the models available are from Cochlear Limited, MedEl Corporation and Advanced Bionics.

**Cochlear Limited Implant Systems [**6**]**

The first commercial model of the company, the **Nucleus 22**, was first marketed in 1985. It was a multichannel device with 22 electrodes. The sound processor, named WSP (Wearable Speech Processor) had a SPEAK processing strategy.

The next generation appeared in 1997 and was called **Nucleus 24**. It had 22 active electrodes and 2 extracochlear reference electrodes. The sound processor, Sprint, disposed of three processing strategies: SPEAK, MSPEAK and CIS. Another novelty was the amovible magnet in cases in which the patient needed to undergo a MRI examination. Also, with this model, the possibility of undertaking neural response telemetry appeared.

In 1999, the company launched the **Nucleus 24 Contour**. The big difference was the precurved electrode array, which allowed the perimodiolar placement inside the cochlea. The sound processor, Esprit, was updated with a fourth coding strategy, ACE and was BTE (behind-the-ear) like a conventional hearing aid, which made a big difference compared to the body-worn processors which existed before. The fourth and fifth generation of sound processors, Esprit 22 and Esprit 3G, were marketed in the year 2000 and 2002 respectively, the last being compatible with all the previous implant models and the first processor with FM capabilities. Regarding the development of the electrode array, the company launched, in 2002, an internal component with a double array, to be used in cases of ossified cochlea.

The next model, named **Freedom**, appeared in 2005 and had two types of electrode arrays: straight and precurved. The last, Contour Advance, had a soft tip to avoid intracochlear insertion trauma. The sound processor had the four already in use strategies and for more: HiAce, Beam, ADRO (for music) and Whisper (for soft sounds). 

In 2008, the company launched the **Hybrid** model, which combined the cochlear implant capabilities with the features of a conventional hearing aid, being intended for the patients with residual hearing.

The next model was marketed in 2009 and it was named **Nucleus 5**. The sound processor could be accessed through a remote control, for easier programming.

The current model available is the **Nucleus 6**. It has two types of sound processors, one with standard and one with compact rechargeable batteries pack. They can both combine the acoustic stimulation capability and are available with five different types of electrode arrays: Straight, Contour Advance, Slim Straight, Hybrid L24 and Double Array. The sound processor can be accessorized with the Aqua+ Coil and silicone sleeve, which makes it waterproof for use during swimming. This latest model represents the sixth generation of internal component and the tenth generation of sound processor.

**MedEl Corporation Implant Systems [**7**]**

The first model of the company, named **Comfort CI**, was marketed in 1989. Its internal component had a ceramic casing and a 4-channel electrode array. The sound processor used an analogue processing strategy. The sound processor was updated in 1991 with a behind-the-ear model.

In 1994, a new model was launched; **Combi 40**, with 8 channels, and a year later the new sound processor, named CIS PRO+ appeared. The development was concentrated around the electrode array, which had a length of 31 mm, to stimulate the entire length of the cochlea.

The next model was **Combi 40+**, also with a ceramic casing, but with 24 active electrodes. The internal part was only 4 mm in depth, being the smallest available at that moment. Another novelty of the model was the MRI-compatibility for up to 1.5 Tesla without the need of surgical removal of the magnet. A year later, the company launched a double array, named Split Electrode, for the ossified cochlea.

In 1999, the sound processor Tempo+ was upgraded to a modular model, the only one available on the market, intended for the use with infants, and was the first BTE of the company.

In 2004, the company launched the **Pulsar** system, which came with four types of electrode arrays: Standard, Medium, Compressed and the Split Electrode. The sound processor, Duet, appeared a year later and had acoustic stimulation capabilities.

The year 2006 brought a new cochlear implant system, **Sonata**, the first model of the company with a silicone casing, and a new generation of electrode arrays, named Flex, for atraumatic insertion. The sound processor Opus, had three coding strategies and improved electronics. The 2009-launched Duet 2 processor had improved acoustic capabilities.

In 2011, the **Maestro** system was marketed; it had 5 types of electrode arrays and two sound processors: a classic one with four coding strategies and an all-in-one named Rondo, which combined the command unit, antenna, battery and microphone in one single casing.

The latest model, named **Synchrony**, was launched earlier that year. It could associate the already in use Rondo processor or a BTE processor named Sonnet, which was waterproof. The company also created an accessory for the Rondo processor to make it waterproof. Another novelty was the MRI-compatibility of up to 3 Tesla.

**Advanced Bionics Company [**8**]**

The first model was marketed in 1996. It was named **Clarion** and it had a ceramic case of the internal component, a precurved electrode array with 8 electrodes and a body-worn sound processor with two coding strategies. A year later, the sound processor was upgraded with a new model, the S-Series, which counted four coding strategies and rechargeable batteries.

The next model, **Clarion II**, was launched in 2001. It had two types of sound processor, one body-worn and one BTE, and a new type of electrode array, the HiFocus, precurved and with 16 active electrodes.

In 2003, the company marketed the **HiRes 90k**, their first model with a titanium casing. The sound processor was named Auria, which used the HiResolution coding strategy.

In 2006 a new sound processor named Harmony was launched; it contained the HiRes Fidelity 120 coding strategy.

The latest model is **Naida CI Q70** system. The implantable component has three types of electrode arrays, each with 16 active electrodes. It can also have a second sound processor, Neptune, which is waterproof.

Lately, all of the latest four sound processors (Auria, Harmony, Naida and Neptune) have come in modular variants: behind-the-ear, some-on-the-ear and nothing-on-the-ear.

## Conclusions

Cochlear implant companies have gone to great length to develop this kind of technology. The Cochlear Company has 7 generations of implantable components and 9 generations of sound processors. The MedEl Corporation has 7 generations of implantable components and 10 generations of sound processors. And Advanced Bionics has developed 5 generations of implantable components and 8 generations of sound processors. These advancements, made in the last 30 years, were made by an evolving technology and by a close collaboration between engineers, surgeons and audiologists. The technology evolved from ceramic and titanium to silicone casing, from 8 to 16 or 24 active electrodes, from big, body-worn sound processors to single-unit or BTE. The milestones of the development of the cochlear implant as it is today were the evolution of coding strategies, the miniaturization of electronic components and the constant development of computer science that allowed the evolution of sound processors with larger processing capabilities.
